# The quagga mussel, *Dreissena rostriformis*: a novel model for EcoEvoDevo, environmental research, and the applied sciences

**DOI:** 10.3389/fcell.2024.1531560

**Published:** 2025-01-09

**Authors:** Andreas Wanninger, Gudrun Schwarze

**Affiliations:** Department of Evolutionary Biology, Unit for Integrative Zoology, University of Vienna, Vienna, Austria

**Keywords:** bivalve, mollusk, evolution, development, evodevo, invasive species, hox, biomineralization

## Abstract

Bivalve mollusks are globally distributed in marine and freshwater habitats. While exhibiting a relatively uniform bodyplan that is characterized by their eponymous bivalved shell that houses the soft-bodied animal, many lineages have acquired unique morphological, physiological, and molecular innovations that account for their high adaptability to the various properties of aquatic environments such as salinity, flow conditions, or substrate composition. This renders them ideal candidates for studies into the evolutionary trajectories that have resulted in their diversity, but also makes them important players for research concerned with climate change-induced warming and acidification of aquatic habitats. Some species, such as the blue and Mediterranean as well as the zebra and quagga mussels, form biodegradable fibers, the byssus threads. These have significant potential for biomimetic approaches by aiding in developing sustainable textiles and other fiber-based fabrics. Despite this broad span of scientific relevance, bivalves remain dramatically understudied and key resources such as high-quality genomes and developmental transcriptomes in combination with established laboratory protocols to carry out state-of-the-art molecular and morphological studies are only available for less than a handful of species. Here, we report on one of the best-investigated bivalves in this respect, the quagga mussel, *Dreissena rostriformis*, an invasive freshwater species. We summarize the current state of knowledge and available resources that make the quagga mussel highly amenable for studying adaptive mechanisms for life in hypoosmotic environments, biomineralization, biomimetics, and evolutionary developmental biology. We argue that the unique combination of biological features and the broad relevance of the quagga mussel for the basic and the applied sciences as well as for biomonitoring and conservation biology measures call for intensified research efforts using *Dreissena rostriformis* as a model.

## 1 Introduction

The phylum Mollusca boasts approximately 120.000 extant species, which makes it the second-most speciose phylum after Arthropoda (Ponder et al., 2019). Its eight class-level taxa, the vermiform, cylindrical Neomeniomorpha (Solenogastres) and Chaetodermomorpha (Caudofoveata), the dorso-ventrally flattened Polyplacophora with eight dorsal shell plates, the univalved Monoplacophora with a cap-like shell, the dorso-ventrally elongated Scaphopoda with a calcareous tube, as well as the more commonly known Gastropoda (snails and slugs), Bivalvia (mussels, clams, oysters, scallops, among others), and Cephalopoda (nautiluses, squids, octopuses) exhibit a vast diversity of phenotypes with adaptations to virtually all terrestrial and aquatic habitats. As a consequence of this morphological and ecological plasticity, numerous features of a proposed ancestral mollusk have either been modified (e.g., the foot, which may function as a creeping, attachment, burrowing, or swimming organ), were entirely lost (e.g., the radula in bivalve mollusks or the shell in various lineages), or have secondarily acquired considerable complexity (e.g., the brain in cephalopods) in a number of representatives (Wanninger and Wollesen, 2019). Accordingly, these morphological variations and the multiple conquest of various habitats render mollusks an ideal group for comparative studies into the developmental and molecular mechanisms that underlie the establishment of these features on a micro- and macro-evolutionary scale.

Despite this huge potential, relatively little is known concerning the evolutionary transitions that have occurred between (and along) the various molluscan class-level lineages. This is partly due to the still poorly resolved interrelationships of the various classes, but even more so, because well-established model systems are scarce and largely lacking for most clades, including prominent representatives such as the gastropods, cephalopods, and bivalves. However, an increasing number of genomic resources have become available across Mollusca over the past few years, which may form an important base for future evolutionary studies (see, e.g., Wollesen and Wanninger, 2023).

Among the shelled groups (Conchifera), Bivalvia constitutes a taxon of particular wide distribution in freshwater and marine habitats. Several bivalve representatives are of outstanding economic importance as source of protein (food) or jewelry (pearls), and are thus prominently featured in aquaculture systems worldwide. On the other hand, being capable of attaching themselves, during settlement and metamorphosis, to hard substrates, either reversibly via so-called organic byssus threads (e.g., blue mussels, zebra and quagga mussels) or permanently via calcareous secretions (e.g., oysters), they constitute major biofouling organisms that pose technical and/or economical problems by, e.g., clogging water pipes or by increasing the water resistance of ship bodies, thereby causing excess fuel expenditure (Inoue et al., 2021; Karatayev and Burlakova, 2022; McCartney et al., 2022; Simmons et al., 2023). Some species (shipworms) may bore into wooden structures and thus contribute to severe damage to estuarine plants as well as manmade constructions including underwater archeological sites (Paalvast and van der Velde, 2011; Hernández and Angelini, 2019).

The bivalve adult bodyplan is unique insofar as bivalves have evolved a number of unique morphological features. Their body is encased by a bipartite shell that is underlain by the mantle epithelium in which the visceral organs of the animal are housed. Studies into the morphogenesis and gene expression during shell formation have found that in the earliest phases of the process the primordial bivalve shell (the protoconch I or embryonic shell) forms from a single shell field, similar to their conchiferan kin. Ontogenetically, the protoconch I is followed by the protoconch II (the larval shell) that, similar to its successor, the teleoconch (adult shell), is formed after metamorphosis by continuous secretion from cells of the mantle margin. Accordingly, the larval and adult shells often show growth patterns, and especially the latter may exhibit highly elaborated ornamentation and coloration (Wanninger and Wollesen, 2019).

In bivalve shells, both lateral halves (valves) are interconnected dorsally by a hinge-like structure and, importantly, by the so-called ligament that is of organic composition. The kinetic energy, that is generated when the animal closes its valves by means of contraction of its prominent adductor muscles, is released upon adductor relaxation, thereby opening the shell passively.

A number of bivalve internal features are characterized by having a more “simple” morphology than the respective counterparts of their conchiferan relatives. A distinct “head” is missing, alongside with the mollusk-specific radula, a rasping tongue that is otherwise present in almost all mollusk species. The nervous system shows some degree of ganglionization along the pedal and visceral nerve cords, but is much less elaborated as, e.g., in gastropods or cephalopods (Ponder et al., 2019).

The bivalve life cycle usually begins with shedding of the male and female gametes into the water column where fertilization occurs (but brooding is also common in some groups). Subsequent development often includes a (usually short-lived and non-feeding, i.e., lecithotrophic) trochophore-type larva that is followed by a planktotrophic veliger larva that may live in the plankton for months. The veliger larva gradually forms most internal organ systems also present in the adult animal (heterochrony). Thus, upon settlement and metamorphosis, where larval features such as the ciliated swimming and feeding device (the velum) and excretory system (protonephridia) are reduced, the young bivalve is immediately ready to commence its benthic lifestyle. Deviations from this ancestral life cycle are found in, e.g., some freshwater taxa (e.g., unionids) that brood their larvae which, upon release and a short swimming period, parasitize on fish skin and gills to which they attach by specific devices such as hooks and threads (Ponder et al., 2019; Wanninger and Wollesen, 2019).

While the vast majority of bivalve species are marine, several lineages have independently ventured into the freshwater realm, the majority of which belonging to the Paleoheterodonta. Some species have shown an astounding capability to cope with fluctuations in salinity values and thus are found in marine/brackish as well as freshwater environments. One such group are the dreissenids, in particular two congeners, *Dreissena polymorpha* (the zebra mussel) and *Dreissena rostriformis* (the quagga mussel), that have an origin in the brackish waters of the eastern European Ponto-Caspian region (Heiler et al., 2013; Aldridge et al., 2014; Calcino et al., 2019; Karatayev and Burlakova, 2022). From there, they spread into the Great Lakes of North America and towards middle and northern Europe from the 1980s onwards, probably by originally travelling with the ballast water of cargo ships (Dahlberg et al., 2023). Once introduced into a new ecosystem, both species may spread rapidly. This is due to the particularly high number of eggs (hundreds of thousands) that may be produced by gravid females and that are released into the water column, where they are fertilized and commence their ancestral, indirect life cycle via free-swimming trochophore and veliger larvae. In favorable conditions, these may stay in the plankton for several weeks and may thus travel considerable distances before they eventually settle and start their benthic lifestyle. Once metamorphosed into a juvenile, dreissenids are high-throughput filter feeders that remove considerable amounts of organic matter, in particular phytoplankton, from the water column, thus impoverishing the food supply for other consumers such as fish and crustacean larvae (Dahlberg et al., 2023). Lacking these primary producers, the water becomes more transparent and penetrable for light, resulting in enhanced growth of submerged plants including algae, thereby significantly impacting the ecosystem’s nutrient and energy fluxes and, ultimately, its species composition. Accordingly, *Dreissena polymorpha* and *Dreissena rostriformis* can be regarded as major “ecosystem engineers”, also because they may form dense aggregates, so-called mussel beds, that serve as habitat for other invertebrate species. In order to minimize their impact on the native freshwater fauna and flora, millions of dollars are invested annually in the United States in various projects that aim at monitoring and controlling the dispersal of these species (Dahlberg et al., 2023).

In addition to their high filtration capacity and reproduction as well as dispersal rate, the zebra and quagga mussel have evolved physiological and cellular mechanisms that allow them to rapidly adapt to salinity conditions that are significantly below that of their region of origin (Calcino et al., 2019). All these factors, that act in favor of *Dreissena*, have contributed to a severe decline of the native freshwater bivalve fauna, particularly of unionids (e.g., the alewife floater, *Anodonta implicata*, its congener, *Anodonta anatina*, the Eastern pearl mussel, *Margaritifera margaritifera*, or the tidewater mucket, *Atlanticoncha* (formerly *Leptodea*) *ochracea*) in North American and European freshwater systems. This has pushed dreissenid bivalves into the limelight of conservation biologists, but up to now, efforts to control these invasive species have proven only little successful.

Given the combination of conserved (typical bivalve bodyplan with shells, filter feeding, reversible attachment mechanisms, free spawning, indirect development in the water column) and derived, i.e. *de novo* evolved (“innovative”) features (e.g., rapid adaptation to low salinity levels), the common zebra mussel (*Dreissena polymorpha*) and quagga mussel (*Dreissena rostriformis*) are prime candidates for research into various biological disciplines including evolutionary developmental biology (evodevo), physiology (adaptation to changes in osmolarity), ecology (invasiveness), and biomineralization, also in the light of climate change scenarios with increased water temperatures and decreased pH values and associated impacts on biocalcification (Calcino et al., 2019; McCartney et al., 2022; Zieger et al., 2022; Simmons et al., 2023).

In the past decade, significant progress in our understanding of dreissenid ontogeny on the morphological and the molecular level has been made. This includes the establishment of protocols for immunofluorescence staining, gene expression studies, and single cell RNA sequencing, as well as the production of a high quality genome and a series of transcriptomes for all key developmental stages of the quagga mussel, *Dreissena rostriformis* (Pavlicek et al., 2018; Calcino et al., 2019; Salamanca-Diaz et al., 2021; Salamanca-Diaz et al. 2022a; Salamanca-Diaz et al. 2022b; Schulreich et al., 2022; Zieger et al., 2022). These resources form an ideal base for tackling biological questions using the quagga mussel for studies from the molecular through the cellular to the organismal and ecosystem scale. Accordingly, topics relevant for the basic sciences (evolutionary biology) as well as applied disciplines (avoidance of biofouling, ecosystem protection) can be addressed using this system. Here, we highlight the state-of-the-art of dreissenid research using the quagga mussel, *Dreissena rostriformis*, as a model, with a focus on its bearing on ecoevodevo studies, summarize and discuss recent findings, and provide an outline for future research paths. We also point out how such data may prove relevant for research into the applied sciences, ecosystems research, climate change, and aquatic pest management.

## 2 *Dreissena* as a model for evodevo research: morphological and molecular aspects of bivalve bodyplan and life cycle evolution

Mollusca is a member of the superclade Lophotrochozoa and its members are characterized by having derived from a last common ancestor with a spiral cleavage pattern (Spiralia). Spiral cleavage has been conserved in most early branching lineages of the various mollusk subclades including Bivalvia. This mode of cleavage is highly determinate, i.e., the fate of each blastomere is predictable, defined early on in development, and conserved across closely related species (Wanninger and Wollesen, 2015; Wanninger and Wollesen, 2019). As such, comparative analyses into the contributions of each blastomere lineage to individual morphological traits allows for insights into the shared ancestry (homology) of given characters, such as ciliated swimming/feeding structures (e.g., prototroch, velum, telotroch), neural and sensory systems (e.g., the larval apical organ), or the shell (see below).

**FIGURE 1:**
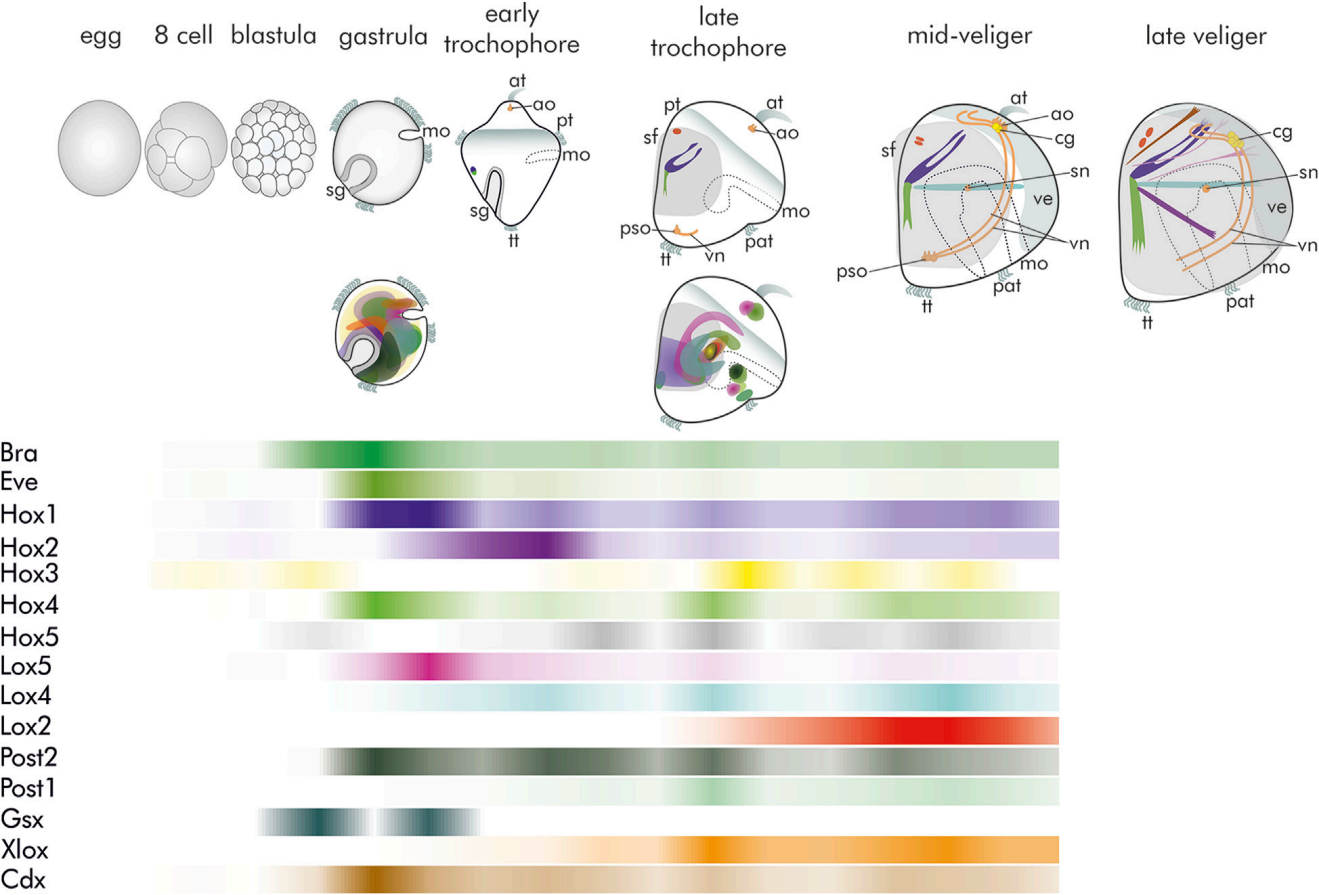
Schematic summary of morphogenesis, gene expression domains, and relative quantitative occurrence of transcripts of *brachyury*, *even-skipped*, as well as the genes of the Hox and ParaHox cluster in *Dreissena*. Data from Salamanca-Díaz et al. (2021) and Schulreich et al. (2022). Top row: Morphogenesis with developing nervous system and musculature. All images are in lateral view with apical facing upwards. (at) apical tuft, (ao) apical organ, (cg) anlagen of the future cerebral ganglion, (mo) mouth opening, (pat) pre-anal tuft, (pso) posterior larval sensory organ, (pt) prototroch, (sf) shell field, (sg) shell gland, (sn) stomatogastric nerve cell, (tt) telotroch, (ve) velum, (vn) ventral neurites. Grey surfaces mark the developing shell and prototroch/velum, respectively. The first serotonin-like immunoreactive (lir) flask-shaped apical cell (orange) appears in the trochophore stage. Myogenesis (green/blue) starts in close proximity to the shell gland. In the late trochophore, two serotonin-lir flask-shaped cells are present in the apical organ, together with the first-formed serotonin-lir flask-shaped cell of the posterior larval sensory organ and the anlage of the ventral neurites. In addition, the dorsal velum retractor (blue), larval retractor (green), and anterior adductor anlage (red) emerge. Four flask-shaped cells form the apical organ in the D-shaped mid-veliger stage, while two roundish, non-sensory cells (yellow) form the anlage of the future cerebral ganglia. In the posterior larval sensory organ, a third flask-shaped cell emerges. A stomatogastric nerve cell is present. The larval retractor (green), the two-partite anterior adductor (red), the dorsal velum retractor (blue), and the ventral velum retractor (turquoise) are present. The immunoreactive cells of the posterior larval sensory organ have disappeared, and the anlage of the future cerebral ganglion has enlarged. Additional muscles of the late veliger larva include two mantle retractors (pink), a foot retractor (purple), and an accessory velum retractor (brown). Middle: Schematic representation of gene expression domains of *brachyury* (*bra*), *even-skipped* (*eve*), Hox, and ParaHox genes. Most genes are expressed in the gastrula and late trochophore stage. Note the overlapping, non-staggered (non-collinear) mode of expression of Hox genes. For color code see bars in relative expression timeline chart below. Bottom: Schematic representation of relative quantitative abundance of *brachyury* (*bra*), *even-skipped* (*eve*), Hox, and ParaHox gene transcripts.

Similar to many of their marine kin, *Dreissena* species have a conserved life cycle during which mature egg and sperm are shed freely into the water column where fertilization takes place. Cleavage is spiral and embryos start to actively swim by means of their cilia as early as in their gastrula stage (Meisenheimer, 1899; Meisenheimer, 1901; Pavlicek et al., 2018; Salamanca-Diaz et al., 2021). This stage is characterized by two ectodermal invaginations, the blastopore (the future mouth) that marks the ventral region, and the more prominent shell field that is part of the dorsal ectoderm (Figure 1). Subsequently, a short-lived planktonic trochophore larva is formed, followed by a veliger larva that may reside in the plankton for several weeks. Development of serotonin-like immunoreactive compounds of the nervous system starts with one flask-like receptor cell in the apical organ of early trochophore larvae (Figure 1; Pavlicek et al., 2018). In the veliger stage, this number increases to four. At the same time, the neurons of the paired visceral cord form, together with receptor cells of the posterior sense organ, a structure that is typical for some larval bivalves, and subsequently the first cells of the future cerebral ganglion emerge. Prior to metamorphosis the apical tuft and the underlying flask cells are gradually lost, as is the posterior sense organ, while the cerebral ganglion is continuously elaborated (Figure 1; Pavlicek et al., 2018).

Similar to neurogenesis, muscle formation starts in the trochophore stage, but elaborate and functional muscles are first found in the veliger larva. The larval myoarchitecture of *Dreissena* is highly complex and includes a number of retractor muscles that enable retraction of the larval body including the velum into the shell. Except for parts of the adductor system, that start forming in the veliger stage, all these larval muscle systems are lost during metamorphic body plan remodelling and all adult systems arise *de novo* (Figure 1; Schulreich et al., 2022).

Data on developmental gene expression are scarce for bivalves, with *Dreissena rostriformis* being one of the best-investigated species today. Ten of the 11 predicted molluscan Hox genes are present in the *Dreissena rostriformis* genome (*Hox-7* appears to be absent). The three bilaterian ParaHox genes were also found (Salamanca-Diaz et al., 2021). Both, the proposed Hox and the ParaHox cluster appear to be broken in *Dreissena*. Compared to other mollusks, Hox gene expression appears unique in the quagga mussel in that no traces of a staggered mode of expression is present. Instead, Hox gene expression, that starts already in the gastrula stage for most genes, shows significant overlap of their expression domains (Figure 1; Salamanca-Diaz et al., 2021). These become largely confined to mesodermal regions, but, again different to other conchiferans, their spatial expression cannot be assigned to any specific developing morphological structures. *Hox-1* is one of the very few exceptions, as it is expressed in the shell field, similar to all other conchiferans investigated so far (Salamanca-Diaz et al., 2021). Since Hox genes are key players in anterior-posterior patterning, the compact, widely overlapping domains in *Dreissena* may be a result of the low degree of longitudinal regionalization of bivalves, whose bodyplan is characterized by the loss of regionally specific traits such as a head or radula. To this end, comparative data on distant but also close dreissenid (marine) relatives within Imparidentia such as myoid, venerid or cardiid representatives would be useful to further assess this hypothesis. However, since protocols to perform functional genetic studies such as RNAi (gene knockdown), gene targeting (gene knockout), or gene editing (using the CRISPR/Cas system) are currently not available for *Dreissena* or any other bivalve, a final argument concerning the function of specific genes remain speculative at present. Yet, the great variety of expression domains of Hox genes across conchiferan representatives suggests that these developmental transcription factors may have been co-opted independently into different functions in the various molluscan lineages, an exciting hypothesis that remains to be tested employing functional genetic methods.

## 3 Biomineralization and the evolution of mollusk skeletal hardparts

Numerous mollusks are characterized by the possession of external armor that my come in spicules (in the aplacophorans), plates (polyplacophorans), or shells (conchiferans). Reductions and modifications of the ancestral conchiferan single-partite, external shell have occurred multiple times along various lineages, having led to complete loss (e.g., in interstitial or terrestrial slugs), internalization (coleoid cephalopods), or bipartioning (some sacoglossan gastropods, most bivalves) in certain representatives (Wanninger and Wollesen, 2019; Ponder et al., 2019).

In taxa that exhibit the ancestral, indirect life cycle, the shell field, that in bivalves may develop as early as in the gastrula stage, initially invaginates (see above and Figure 1). Subsequently, the shell field evaginates (“flattens”) and secretes the organic matrix (the periostracum) that serves as a scaffold for the calcification process during which calcium carbonate molecules are deposited to eventually produce the embryonic shell (protoconch I) (Wanninger and Wollesen, 2015). The protoconch I appears to calcify from two centers of the originally single shell field, thereby establishing the bipartite condition of the bivalve shell. Calcification of the protoconch I is rapid, resulting in the smooth appearance of its surface. In some long-lived planktonic gastropod larvae and numerous bivalves (including *Dreissena*), an intermediate larval shell (protoconch II) is formed that, similar to its successor, the teleoconch (the adult shell), is continuously secreted from cells of the mantle margin. Thus, while the larval shell (where present) forms an ontogenetic continuum with the adult shell, the fundamentally different mode of embryonic shell formation calls into question the putative homology of the latter with the former two shell types (Salamanca-Diaz et al., 2022a).

Recent studies employing singe cell RNA sequencing have shown that the gene complement of both, embryonic and adult shells, in bivalves include a large number of (taxonomically restricted?) genes that show no ortholog match with other metazoans (Salamanca-Diaz et al., 2022a). Accordingly, these genes may thus be prone to high mutation rates over short periods of time, a characteristic of so-called “rapidly evolving” morphological features (Jackson et al., 2006; Kocot et al., 2016; Aguilera et al., 2017; Clark et al., 2020; Yarra et al., 2021; Salamanca-Diaz et al., 2022a). A comparison of the genes expressed during embryonic and larval shell secretion further revealed that both ontogenetic shell types differ greatly in the suite of genes expressed during their secretion, lending support to the hypothesis that the first-formed embryonic shell is not homologous to the larval shell (and thus likely neither to the adult shell; Salamanca-Diaz et al., 2022a). However, the comparative database of gene expression during the formation of all three bivalve shell types is very scant, thus hampering well-founded conclusions into the evolutionary relatedness of these three shell types to each other.

The plasticity of bivalve skeletogenesis is not only manifested by the highly diverging genes that appear to be involved in the process, but also by the morphological properties exhibited by their shells. Accordingly, these may come in various, differing layers that build the shells in the respective lineages, variations in their colors and color patterns, and, most strikingly, in innumerous modifications of their calcareous surface that may include various ornamentation such as ribs, spines, and the like (Ponder et al., 2019). A particularly striking example includes the teredinids (shipworms) that have secondarily transformed their shell valves from a protective apparatus into a drill-like boring device. Given the fact that all bivalves derive from a last common ancestor with a bivalved, most likely small, simple, and little elaborated shell, further comparative analyses into the molecular and genetic toolkit on the DNA (genes), RNA (gene expression), proteome (shell matrix components), and biomineralization (e.g., ions incorporated in the various shell layers) level will offer a wealth of opportunities to assess evolutionary processes and key drivers on a (micro)evolutionary level - also outside Bivalvia, Mollusca, or even Bilateria, since the ability to form mineralized exo- or endoskeletal features dates back well into the Pre-Cambrian (Murdock, 2020).

**FIGURE 2:**
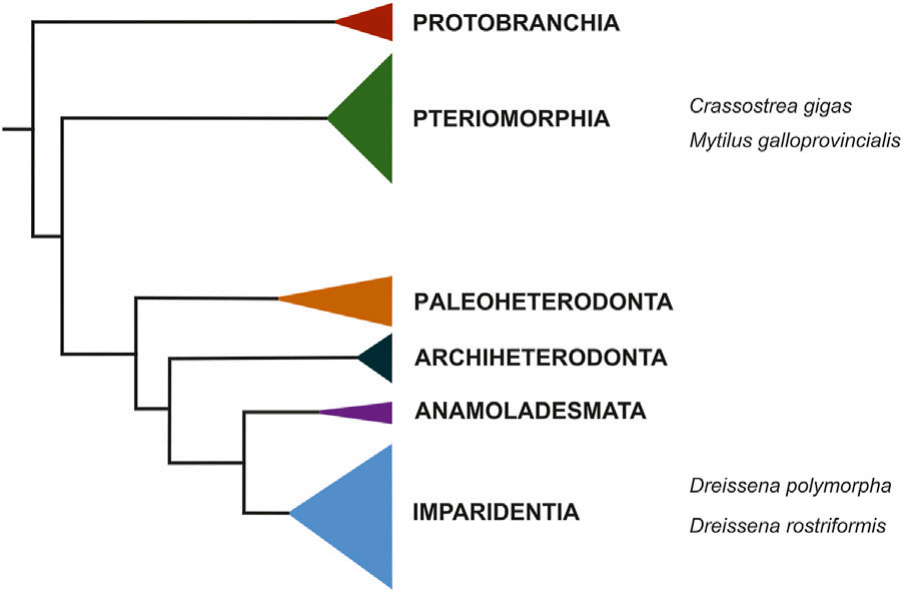
Condensed phylogeny of Bivalvia based on a supermatrix composed of 47 molluscan taxa. Modified after Calcino et al. (2019). The quagga mussel, *Dreissena rostriformis*, together with its congener *Dreissena polypmorpha*, the zebra mussel, is nested within Imparidentia. The only two other bivalve species for which comparable genomic and transcriptomic resources are available, the Pacific oyster, *Crassostrea gigas*, and the Mediterranean mussel, *Mytilus galloprovincialis*, are marine species and are phylogenetically distant to the quagga mussel, residing within Pteriomorphia.

In addition to research agendas revolving around evolutionary questions, the analysis of biomineralization processes, particularly in aquatic representatives, has become increasingly popular over the past decades because it provides the possibility to directly observe putative consequences of various issues dealing with the topic of climate change impact on living organisms. As one of the most abundant and ecologically as well as economically important biomineralizing marine animals with billions of offspring produced yearly, bivalves are ideally suited for research into the consequences that, e.g., lower pH values (acidification) may have on the process of producing mineralized skeletal elements using standardized laboratory settings. Numerous studies have proven that continued acidification of aquatic environments may severely impact the vital process of biomineralization in various marine and freshwater invertebrates, particularly gastropods and bivalves, thereby posing significant threats to the survival of species that rely on functional processes to build their skeletal features (e.g., Fabry et al., 2008; Lombardi et al., 2011; Bray et al., 2014). However, despite these well-known negative consequences of acidified habitats for their inhabitants, very little is known about the molecular and genetic mechanisms underlying these processes. To this end, high-quality genomes in combination with tight series of developmental transcriptomes by RNA sequencing hold a strong potential to gain insights into the dynamics and key mediators of biomineralization, since such resources can be specifically mined for suspected candidate genes. Potential hits can then be further analyzed using *in situ* hybridization to assess the tempo-spatial expression of these genes in the organism of interest across its developmental timescale and verify if the respective gene is indeed active in tissue with biomineralization properties. Such tight series of developmental transcriptomes do exist for only a few bivalve species, e.g., the quagga mussel, *Dreissena rostriformis* (Calcino et al., 2019), the Mediterranean mussel, *Mytilus galloprovincialis* (Gerdol et al., 2020; Miglioli et al., 2024), and the Pacific oyster, *Crassostrea gigas* (Zhao et al., 2018; Peñaloza et al., 2021). While the latter two belong to the Pteriomorphia, *Dreissena* species are part of a distant bivalve lineage, the Imparidentia (Figure 2), and thus are key for comparative evolutionary studies, including those that aim at reconstructing the processes and players behind the formation of mineralized hardparts. Such data should render more reliable predictions as to how further (anthropogenically induced) environmental change may have a negative bearing on animals that rely on the formation of skeletal elements, thereby aiding in enforcing protection measures in the future (see below).

## 4 The molecular and cellular foundations of freshwater adaptation in the quagga mussel

There is little doubt that life on Earth evolved in marine environments. From there, animals have ventured onto land and into freshwater numerous times independently. These transitions evoked dramatic physiological adaptations with respect to change in diets, metabolism, excretion, and osmoregulation. In particular, desiccation and osmoregulatory issues need to be overcome when exchanging the high osmotic marine for the hypoosmotic freshwater or the dry terrestrial realm.

**FIGURE 3:**
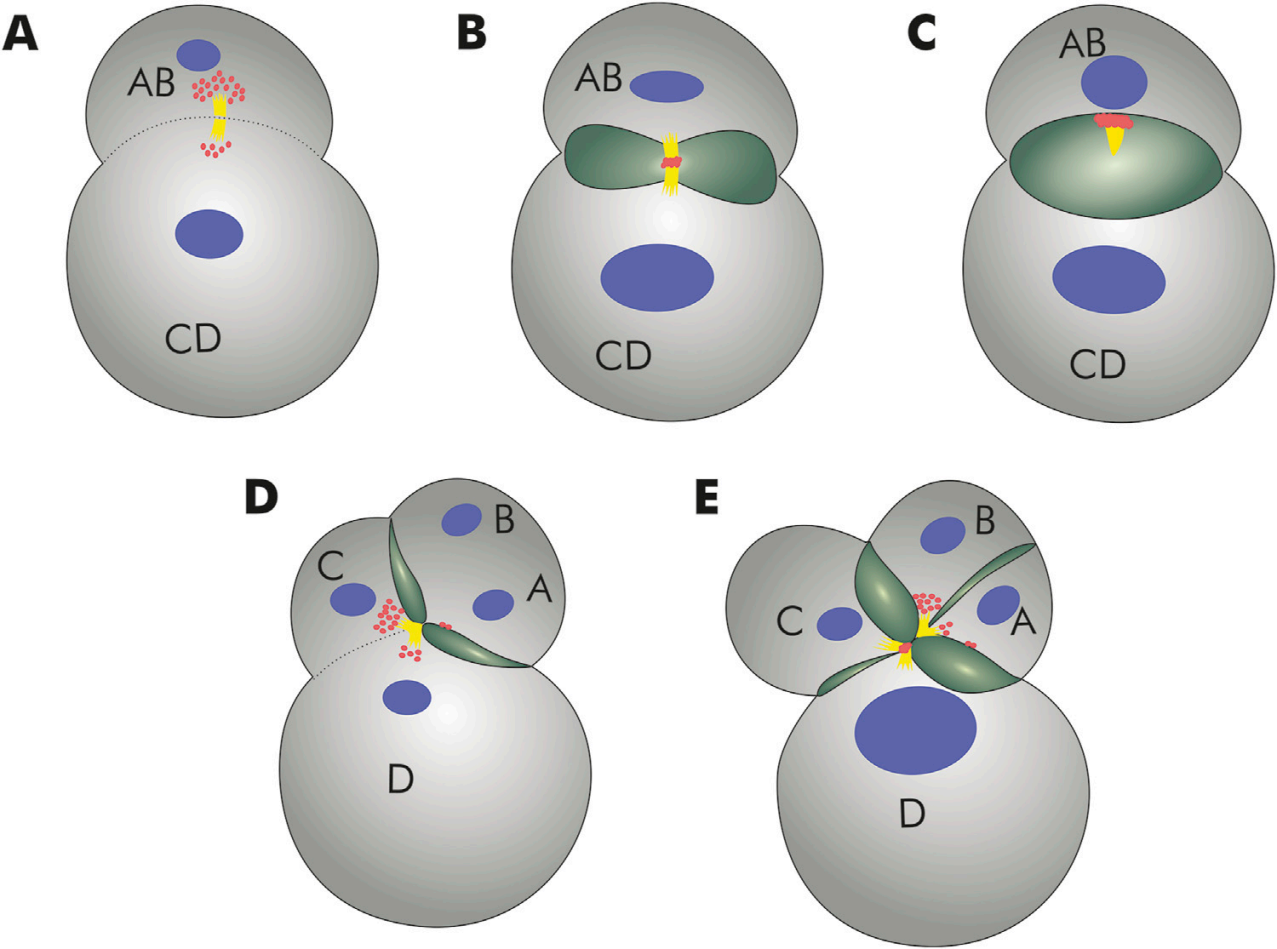
Schematic representation of lumen formation during *Dreissena rostriformis* cleavage as proposed by Zieger et al. (2022). **(A)** Aquaporin (red) accumulation at the cytokinetic bridge (yellow). Cell nuclei in blue. **(B)** Aquaporin is recruited to the midbody during cleavage. Cleavage cavity (green) formation is initiated. **(C)** Expansion of the cleavage cavity and separation of the blastomeres AB and CD. One of the two daughter cells inherits the aquaporin immunoreactive midbody remnant. **(D)** The larger CD blastomere divides slightly earlier than the smaller AB blastomere, giving rise to a transient three-cell stage. Note the aquaporin accumulation prior to formation of the second midbody between blastomeres C and D. Two additional cytokinetic bridges are produced by the second round of cleavages; these bridges are displaced towards the interface between the A/D and B/C blastomeres in the middle of the embryo. **(E)** Subsequent cleavages result in additional cavities along the cleavage furrows of forming blastomeres with aquaporin remaining accumulated along the cavity membranes.

Bivalves have invaded freshwater habitats independently on numerous occasions (Calcino et al., 2019). How do they cope with the dramatic osmoregulatory challenges? In adults, excess water is expelled via distinct, multicellular organ systems such as the nephridia. Water removal mechanisms, however, are far less well understood for the early ontogenetic (cleavage) stages, that mark the most vulnerable phase during animal development. It had long been known that *Dreissena* and other freshwater species form large, rhythmically contracting, fluid-filled cavities during early cleavage stages, features that have been attributed to mechanisms of water expulsion in order to avoid cell rupture (Allen, 2000; Tani et al., 2002; Datta et al., 2011; Calcino et al., 2019). Recent combined genomic, cell biological, and developmental studies have shown that a vacuolar ATPase subunit, a sodium/hydrogen exchanger, and a lophotrochozoan-specific aquaporin water channel are involved in the process of water ejection via these cleavage cavities (Calcino et al., 2019). Thereby, a maternally inherited, *Dreissena rostriformis*-specific aquaporin accumulates at the so-called cytokinetic bridge, an arrangement of centrally-located microtubules that forms in the region of the midbody during the process of cell division (Figure 3A; Zieger et al., 2022). During cleavage cavity expansion, the concentration of this aquaporin in the midbody increases (Figure 3B). After cleavage cavity collapse (and extrusion of the contained liquid) as well as separation of the two daughter cells from each other, the midbody and its associated aquaporin is inherited by one of these two cells (Figure 3C). Thereafter, a new cycle of aquaporin recruitment into the midbody region in association with vacuole formation commences once the next generation of cleavage cycles is initiated (Figure 3D; Zieger et al., 2022). While the exact cytological and molecular mechanisms involved in cellular water extrusion remain elusive, these findings on *Dreissena rostriformis* form an important base for future (comparative) research into this matter. The independent multiplication of aquaporin-coding genes in various freshwater bivalve lineages suggests high selective pressures on freshwater-conquering mussels, and putatively also other free-spawning invertebrates, in adopting such an aquaporin-mediated system of water excretion during embryogenesis for survival in hypoosmotic habitats.

## 5 Discussion

### 5.1 Available *Dreissena rostriformis* resources for EcoEvoDevo studies

Alongside *Mytilus galloprovincialis* and *Crassostrea gigas*, *Dreissena rostriformis* is one of the very few bivalve species that are currently at the forefront of becoming “true” model systems in ecological and evolutionary developmental biology. For *Dreissena rostriformis*, a series of developmental transcriptomes from the unfertilized, freshly spawned egg through late larval stages is available, as is a high quality genome (Calcino et al., 2019). Reliable protocols for immunofluorescence staining, electron microscopy, histology, and gene expression analyses are well established for *Dreissena* (Pavlicek et al., 2018; Calcino et al., 2019; Salamanca-Diaz et al., 2021; Salamanca-Diaz et al. 2022a; Salamanca-Diaz et al. 2022b; Schulreich et al., 2022; Zieger et al., 2022), and the cell lineage of key organ systems has long been described (Meisenheimer, 1899; 1901). At least in Central Europe, the reproductive period is long, spanning at least from April through October. Spawning can be induced artificially by the addition of serotonin, and hundreds of thousands of offspring can be harvested per individual spawn. Both, adults and developmental stages are easily kept in the laboratory, thus keeping costs and required manpower for animal care low (Pavlicek et al., 2018; Calcino et al., 2019). However, further research should be dedicated to increase the yield of postmetamorphic individuals. Metamorphosis is a key step in the life cycle of indirect developing animals, and in aquatic invertebrates this process often fails in laboratory cultures, resulting in a high percentage of loss of individuals before the juvenile stage is achieved. While this undoubtedly reflects the condition in the wild (producing thousands of offspring is a strategy to cope with high mortality rates prior to adulthood), research should strive towards establishing laboratory conditions to close the life cycle from gametes to adult to gametes as reliably as possible. This is still in its infancy for bivalves in general, because larval phases are often several weeks long, their optimal diet is still unknown, and the mediators of larval metamorphosis - as for almost all aquatic invertebrates - remain elusive. However, for many ecological, developmental, and evolutionary, as well as applied research agendas, the quagga mussel already today offers a wide range of possibilities that are worth exploring. This is particularly true since, within Bivalvia, *Dreissena* species reside within Imparidentia, a lineage that diverged early from the Pteriomorphia to which the two other promising bivalve models, *Crassostrea* and *Mytilus* belong (Figure 2). Due to this early phylogenetic split, the genetic distance of dreissenids to pteriomorphs is relatively large, an important prerequisite for evolutionary studies on broader scales and across long geological timescales.

With both the quagga and the zebra mussel genomes available (Calcino et al., 2019; McCartney et al., 2022), microevolutionary processes can be studied using both dreissenid congeners in comparative approaches. Both species often co-occur alongside each other, with *Dreissena polymorpha* usually being the primary colonizer of a given habitat. However, despite rapidly forming large and stable cultures, several reports have shown that in some habitats *Dreissena polymorpha* may be outcompeted over time by the quagga mussel, *Dreissena rostriformis*, which may lead to partial or full replacement of the former by the latter (Karatayev and Burlakova, 2022). Since both species strikingly resemble each other with respect to size, morphology, life cycle, ecological, and physiological needs, the drivers that render *Dreissena rostriformis* ecologically more competitive than its congener remain largely elusive. A first step towards answering such questions may lie in in-depth comparative genomic analysis of both species, which may reveal potential differences in genes encoding for metabolic pathways, efficient usage of food resources, adaptive capacities to life in freshwater, or higher tolerance to environmental factors including those related to climate change issues.

### 5.2 Under pressure: the quagga mussel in a changing world

Due to their rather non-selective filter-feeding lifestyle, bivalves in general, and those that form large colonies such as dreissenids in particular, have long been recognised as good candidates to monitor nutrition intake and degrees of pollution, including the accumulation of microplastics, in aquatic environments (Inoue et al., 2021). In the age of genomics, the impact of habitat changes can now be monitored not only on the macroscopic level, but also on the level of genes and signaling cascades that are involved in producing key morphological features throughout the life cycle of the respective species. Accordingly, research into the genetic basis of biomineralization is a field that has received considerable attention over the past decades, and the dramatic consequences of human-made impacts on pH decrease (acidification) on aquatic organisms that depend on forming mineralized hardparts has been widely reported (Fabry et al., 2008; Lombardi et al., 2011; Bray et al., 2014). As important ecosystem engineers, the decline of bivalve populations due to failures in the process of biocalcification, particularly in the vulnerable larval stages, will have strong impacts on limnic ecosystems with significant bivalve (including dreissenid) populations (see above). Direct consequences of dramatically reduced population densities will include decrease in microscopic phytoplankton, the major food source of these efficient filter feeders, ultimately leading to lower yield of commercial fish. The increased transparency of the water column after removal of major parts of the phytoplankton may increase growth rates and depth distribution of aquatic macrophytes, which in turn may lead to greater organic waste production and increased oxygen-poor regions at greater depths. In a way, these processes may (partially) reverse those originally imposed on limnic ecosystems by introduction of these invasive species since the 1980s, but if such impact is desired is a matter of discussion in a larger bioconservation context. In any case, close monitoring of putative defects in larval and embryonic shell formation of dreissenid bivalves will allow for predictions of their putative decrease in a given habitat and associated potential environmental impact.

Aside from calcareous exoskeletal elements, dreissenids, as well as other bivalves, e.g., mytilids, have evolved additional, organic biomaterials that are prone to suffer defects from increased CO2 and thus decreased pH levels. These are the byssus threads, that serve for firm but reversible attachment to hard substrates, allowing the animals to withstand currents or other forms of mechanical displacement. Byssus threads are secreted from the byssus gland in the foot as a liquid proteinaceous substance that hardens once in contact with water. The composition and biomechanical properties of bivalve byssus threads have been a matter of research for some time (see below), and recent data indicate that their functional performance suffers from acidification, just like their calcareous shells (O’Donnell et al., 2013). As such, it has been shown for marine *Mytilus* mussels that, if exposed to higher CO2 regimes that resulted in a change in pH from 8 to 7.5 during secretion, the tenacity of their byssus threads was decreased by 40% (O’Donnell et al., 2013). This occurred largely in the distal-most region of the byssus, where a so-called plaque is formed that attaches to the substrate. Interestingly, these negative effects on byssus performance occurred rapidly in short-time exposure experiments, during which shell and tissue growth as well as other physiological processes remained unaffected, indicating that these attachment structures may suffer faster from acidification events than mineralized structures (O’Donnell et al., 2013). In addition, byssus produced under heat stress have likewise proven to be less stable and exhibit slower secretion rates in marine species (Xu et al., 2023). Whether dreissenid byssus would suffer similar weakened performances in waters with climate change-induced increased temperatures remains unknown, but if so, this might have dramatic effects on their ability to manifest themselves and form stable colonies, particularly in habitats with temporary or permanent turbulence such as smaller streams or rivers, where attachment to the substrate is key to survival of these benthic animals. Accordingly, research focussing on the performance of *Dreissena* byssus threads under predicted future increasingly acidic and higher temperature regimes will allow for modelling the potential of *de novo* colonialization events as well as the stability of existing colonies in given freshwater habitats. Obviously, such environmentally caused reduced tenacity of *Dreissena* byssus threads will also affect the biofouling capacities of these bivalves, and survival rates may generally be significantly lowered due to defects in shell formation.

### 5.3 Use me: *Dreissena* as a model for applied research

Byssus threads have been used by humans for centuries, primary as textiles of the privileged (Ehrlich, 2019). More recently, the ability of bivalve byssus to adhere to substances in wet environments has resulted in the development of a number of synthetic polymers, particularly for biomedical applications, with anchoring, adhesive, and sealing properties (Lee et al., 2011).

While the molecular nature of the *Mytilus* byssus has been known for some time, the detailed protein composition that makes up the dreissenid byssus, in particular that of the quagga mussel, *Dreissena rostriformis* (aka *Dreissena bugensis*), was only recently revealed (Simmons et al., 2023). This study showed that byssus threads of the quagga and zebra mussel are stronger and more resistant than those of *Mytilus* (Brazee and Carrington, 2006). This is probably due to several unique properties of the dreissenid byssus which include the lack of collagen (present in all other bivalve byssi investigated so far) and the addition of a β-sheet crystalline structure that is also found in spider silk (Simmons et al., 2023). In spiders, these β-crystallites contribute to silk strength, and its deployment into the dreissenid byssus may thus be key to producing particularly tenacious threads. A significant drawback in artificial synthesis of such β-crystalline-containing fibers inspired by spider silk has so far been the complex biochemical process by which these fibers are formed, requiring a combination of acidification, ion exchange, applied sheer force, and water removal in the spider (Rising and Harrington, 2023; Simmons et al., 2023). This process, however, appears to be simplified in dreissenid bivalves, since it seems to only depend on mechanical forces generated by the moving foot that houses the secretory byssus gland, rather than additional molecular mechanisms (Simmons et al., 2023). Accordingly, in addition to the biomedical applications based on mytilid byssus components such as aided wound healing, artificially produced fibers employing the unique properties of dreissenid byssus threads will open a whole new dimension of applications for resistant, protein-based, biodegradable and thus environmentally friendly polymer fibers. To this end, artificially produced fibers that mimic *Mytilus* byssus have been shown to be able to replace non-degradable synthetic fibers used in marine applications for, e.g., building enclosures for commercial seaweed and fish farming as well as nets for the fishing industry (Hong et al., 2024). These biomimetic fibers degrade in marine environments by bacterial activity, and thus are highly promising candidates to reduce the waste of nylon and other synthetic plastics in human applications on oceanic systems. Interestingly, byssus-producing mussels are themselves able to displace these fibers in order to move away from their attachment area, but the underlying molecular mechanisms and the genes involved in breaking down these fibers in the mussel remain unknown (Giraldes et al., 2019). It must, however, be assumed that this process is bacteria-independent and rather caused by enzymes produced by the bivalve itself. Accordingly, enhanced research into the molecular pathways underlying not only the secretion, but also the (enzymatic) degradation of bivalve byssus threads using the transcriptomic and genomic resources available for the quagga mussel appears highly promising for developing more sustainable textile production.

### 5.4 The quagga mussel in future EcoEvoDevo research

As pointed out above, the quagga mussel, *Dreissena rostriformis*, holds a large potential for future studies in the applied, environmental, and evolutionary sciences. With the bulk of transcriptomic, genomic, morphological, and developmental data available and their amenities as laboratory system (inducible spawning, large number of offspring), a wealth of questions revolving around the field of evolutionary developmental biology can be approached. For example, the evolution of asymmetry in primarily bilaterian animals is one of the current hot topics in EvoDevo (e.g., Namigai et al., 2014). Several bivalves (e.g., oysters) show strong tendency towards an asymmetric bodyplan morphology, while others, such as the dreissenids, do not. Accordingly, comparison of up- and downregulation of candidate (or novel) genes during establishment of morphological asymmetries with the behavior of the respective homologs in symmetrical reference species such as the quagga mussel can provide important insights into how the deviation from a once common developmental scheme (bilaterality) evolved to eventually produce non-symmetrical offspring.

For assessing the plasticity of gene expression signatures in various shell types within and across molluscan lineages, the database from *Dreissena rostriformis* forms an ideal reference for future comparative research. Likewise, the bivalve attachment system, the byssus, has been shown to show considerable variation with respect to their protein composition, whereby oysters form superhelical nanofibrils (Pasche et al., 2018), the giant clam uses tetrameric coiled coils, and mytilids form their threads from collagen precursors (Waite et al., 1998). Collagen appears to be absent in *Dreissena* species; instead, it seems that dreissenids have acquired gene(s) encoding for the predecessor(s) of the α-helical coiled coils used in their byssus by horizontal gene transfer from associated bacteria (Simmons et al., 2023). These coiled coils may have significantly contributed to the extraordinary tenacity of the *Dreissena* byssus (Simmons et al., 2023). Despite this progress, the detailed gene regulatory networks underlying bivalve byssus formation - and thus the putative convergent evolution of this system along various lineages - remains obscure.

As outlined above, formation of both the biocalcified exoskeletal hardparts and their adhesive threads are key to the success of dreissenids as invasive species. With *Dreissena rostriformis* as a powerful model at hand, there is hope that research into the mechanisms that govern crucial developmental processes such as shell and byssus formation as well as the transition from the larval to the juvenile phase (settlement and metamorphosis) may soon provide us with tools that help to reduce the fouling capacities and the spreading of these animals in non-native waterways and will lessen their negative impact on human-made constructions.

There is a lot to explore using bivalve evolution using the emerging triumvirate of the Mediterranean mussel, *Mytilus galloprovincialis*, the Pacific oyster, *Crassostrea gigas*, and the quagga mussel, *Dreissena rostriformis*, as models. As with every study that aims at decoding evolutionary phenomena, comparative analyses are pivotal in this endeavor. With the quagga mussel as the only freshwater bivalve currently at hand that is amenable for large-scale developmental studies, conventional evodevo research programmes, that are mainly concerned with questions of bodyplan evolution, can now be expanded and broadened towards topics such as the evolution of invasiveness and the adaptation to freshwater environments including the underlying molecular, cellular, developmental, and physiological processes.
